# The effects of genre on the syntactic complexity of argumentative and expository writing by Chinese EFL learners

**DOI:** 10.3389/fpsyg.2022.1047117

**Published:** 2022-11-10

**Authors:** Liping Pu, Renquan Heng, Cong Cao

**Affiliations:** ^1^Soochow College, Soochow University, Suzhou, China; ^2^School of Foreign Languages, Soochow University, Suzhou, China

**Keywords:** genre, syntactic complexity, argumentative compositions, expository compositions, Chinese EFL learners

## Abstract

Genre researchers have found that writing in different genres involves different cognitive task loads and requires different linguistic demands. Generally speaking, narratives involve the description of events with a focus on people and their actions within a specific time frame, whereas non-narrative genres focus on making an argument or discussing ideas or beliefs in a logical fashion, thus resulting in distinct language features. However, the vast majority of genre-based studies have either focused on one single genre or made comparisons between narrative and non-narrative writing (mostly argumentative) in academic contexts without examining how EFL writers perform across non-narrative genres. Moreover, the measures used in quantifying the syntactic complexity of writing are varied, leading to inconsistent findings. This study investigated the effects of genre on the syntactic complexity of writing through comparing argumentative and expository compositions written by Chinese English-as-a-foreign-language (EFL) learners over one academic year. Fifty-two participants were asked to write eight compositions (with two genres alternated), four argumentative and four expository, which were parsed *via* the Syntactic Complexity Analyzer. The results with time as the within-subjects variable showed a significant development of syntactic complexity in both argumentative and expository compositions over one academic year. Meanwhile, the paired-sample *t*-test with genre as the within-subjects variable exhibited a higher syntactic complexity in argumentative compositions than in expository ones on most of the 14 measures examined at four time points over the year. Additionally, a two-way repeated measures analysis of variance with genre and time as independent variables ascertained an interactional effect of time and genre on some of the 14 measures. The present study tested and verified the impact genre exerts on the syntactic complexity of writing, providing implications for teachers to be more informed in teaching and assessing EFL writing and for students to be more conscious of genre difference in EFL writing.

## Introduction

Over the past decades, research has shown that writing in different genres involves varied cognitive task loads (Kamberelis and Bovino, 1999; Beauvais et al., 2011; Bi, 2020) and requires different linguistic demands (Berman and Nir-Sagiv, 2004; Nippold, 2004; Beers and Nagy, 2011; Biber et al., 2016; Xu, 2020; Atak and Saricaoglu, 2021). Generally speaking, genres can be classified into narratives and non-narratives, and non-narratives can be further divided into expository, argumentative, persuasive, and the like. For the most part, narratives involve the description of events with a focus on people and their actions within a specific time frame, whereas non-narrative genres focus on making an argument or discussing ideas or beliefs in a logical fashion (Tian, 2014). To be specific, narrative essays tend to involve more third-person pronouns and use of the past tense; expository essays might contain more use of relative clauses and attributive adjectives in describing the theme; and argumentative essays prefer attributive clauses in demonstrating the statement (Brunner, 1986; Biber and Conrad, 2009; Berman and Slobin, 2013). In writing tasks across genres, English-as-a-foreign-language (EFL) writers are generally faced with a task that can be challenging in two aspects: On one hand, they need to identify the special discourse type in the required communicative context, and on the other hand, they need to utilize all their language resources to craft proper expressions to serve the specific context. For instance, Beauvais et al. (2011) found that students used different writing strategies to meet the demands of different writing genres. Compared to narrative writing, argumentative writing required more cognitive effort. Therefore, the students spent more time planning to write an argumentative text because it required a complex and sophisticated knowledge-transforming strategy.

Considering the differences in genres, teachers should both attach importance to writing complexity and take account of the effects of genre differentiation when assessing students’ proficiency level and determining their developmental trajectories in the target language (L2). Hyland (2007) contended that “effective teaching recognizes the wants, prior learning, and current proficiencies of students, but in a genre-based course, it also means, as far as possible, identifying the kinds of writing that learners will need to do in their target situations and incorporating these into the course” (p. 152). Likewise, when illustrating the criteria for assessing the Chinese “FLTRP Cup” English writing contest, Tian (2014) proposed that due attention should be paid to the differences between expository writing and argumentative writing in that both genres are expected to effectively address the topic and the task, but expository writing should present a clear thesis in a formal style and an objective tone; while argumentative writing should present an insightful position on the issue, and the position should be strongly and substantially supported or argued.

To date, the overwhelming majority of previous empirical studies assessing EFL writing have either focused solely on one single genre or made comparisons between narrative and non-narrative writing in academic contexts without examining how EFL writers perform across non-narrative genres (Qin and Uccelli, 2016). Moreover, in the actual teaching of writing, argumentative writing is generally the preferred option for practicing while other genres of writing are more or less ignored. Therefore, in writing instruction and practice, the need arises for students to have the opportunity to be exposed to different genres of writing and for teachers to provide students with specific and targeted guidance and instruction based on different styles of writing.

The present study, through comparing the syntactic complexity of students’ argumentative and expository compositions, attempts to contribute to the existing literature in two ways. First, by examining how students write in these two genres, we are able to see the changes in syntactic complexity in Chinese EFL learners’ English writing over one academic year. Second, by examining the differences in students’ argumentative and expository compositions, we are able to explore whether genre exerts any effects on syntactic complexity in Chinese EFL learners’ argumentative and expository compositions and, furthermore, if the effects of genre are retained over the course of 1 year.

## Literature review

### Genre in writing

Text genres are primarily divided into narrative and non-narrative in both academic and non-academic contexts (Brunner, 1986). Narratives focus on events and actions in settings performed by the characters, whereas non-narratives (e.g., argumentative, expository, descriptive) focus on ideas and concepts and express the unfolding of claims and argumentation in a logical fashion (Berman and Slobin, 2013). Genre has much to do with cognitive task complexity in relation to two competing hypotheses, namely the cognition hypothesis (Robinson, 2001, 2007) and the limited attentional capacity model (Skehan, 1998). Different genres place different levels of cognitive demands on students, with narrative being the least cognitively demanding, expository cognitively more demanding than narrative, and argumentative the most cognitively complex (Genung, 1900; Bain, 1967; Weigle, 2002). Yang (2014) explored four genres, namely narrative, expository, expo-argumentative, and argumentative, as regards different levels of cognitive demands and reasoning on the attentional resources of participants, finding that the argumentative essays were significantly more complex in global syntactic complexity features than the essays on the other rhetorical tasks. She interpreted her findings by fitting genre into the resource-directing dimension of Robinson’s cognitive hypothesis where reasoning and perspective-taking are involved. However, the 375 subjects were of varied proficiencies, including both English majors and non-English majors, and they were assigned different writing tasks, thus creating confusion as to the real cause underlying the differences.

When it comes to the actual writing, genre differences are reflected in the way words and phrases are selected as well as in the formation and connection of clauses or sentences that best describe their own characteristics (Biber and Conrad, 2009). Since the purpose of this study is to delve into the differences in syntactic complexity between argumentation and exposition, we shall focus on these two genres only and disregard the others herein.

Ruiz-Funes (2015) contended that expository and argumentative genres could be operationalized as the reasoning demands of cognitive task complexity and that they both involve a resource-directing feature of task complexity. Argumentation is a genre that invites a writer to give personal opinions and judgment on a debatable issue or statement and to take a stand on the issue/statement based on facts, generalizations, and reasoning. It is, more often than not, topic-oriented, which requires the writer to impose a logical structure to interrelate ideas in a coherent manner and to organize claims and arguments in a stepwise hierarchical format (Grabe, 2002), where clauses are often used to link ideas and enable complex expressions. By contrast, exposition invites the writer to explain and to provide information about something (not to take a side on something debatable or to argue on the topic) based on facts and generalizations of events and states. The ideas for expository production derive from general world knowledge and academic learning, and expository texts often express the unfolding of claims in containing the theme requirements of noun phrases using nominalizations as clause subjects that condense prior information and present what has already been put forward so that further comment can be made about it (Ravid, 2005).

### Syntactic complexity in writing

In second language research, syntactic complexity can be viewed “as a valid and basic descriptor of L2 performance, as an indicator of proficiency and as an index of language development and progress” (Bulté and Housen, 2014, p. 43). Nonetheless, no consensus has been reached as to how to define the construct of syntactic complexity. In Gaies (1980), syntactic complexity was defined as the ability to express more ideas and more thoughts with the use of fewer words, while Skehan (1996) considered syntactic complexity as more elaborate language and more various sentence patterns. In Wolfe-Quintero et al. (1998), syntactic complexity was taken as the range of forms that surface in language production and the degree of sophistication of such forms, whereas Ortega (2015) stated that “syntactic complexity indexes the expansion of the capacity to use the additional language in ever more mature and skillful ways, tapping the full range of linguistic resources offered by the given grammar in order to fulfil various communicative goals successfully” (p. 82). Similarly, Lu (2011) considered syntactic complexity as the range of syntactic structures produced and the degree of sophistication of such structures, which is the way we chose to define syntactic complexity in the present study.

Syntactic complexity has been generally considered essential in assessing the performance of foreign language writing (Wolfe-Quintero et al., 1998; Housen and Kuiken, 2009; Lu, 2011; Staples et al., 2016; Yoon, 2017; Ana, 2018; Kyle and Crossley, 2018). For this reason, many researchers have attempted in the past decades to establish effective and reliable measures of syntactic complexity to assess language progression and judge the writing level and proficiency of learners (De Clercq and Housen, 2017; Ansarifar et al., 2018; Casal and Lee, 2019; Bi and Jiang, 2020; Kim, 2021; Huang et al., 2022; Li et al., 2022). Ortega (2003) showed cumulative evidence concerning syntactic complexity by assessing university students’ writing performance and overall proficiency in the target language through synthesizing 25 studies, testifying to the use of syntactic complexity as an indicator of writing quality and language proficiency. Guo et al. (2013) contended that we could, to some extent, anticipate language learners’ essay scores by looking at their language characteristics and structures in both integrated and independent writing.

Syntactic complexity is often conceptualized as a multidimensional construct, with each dimension requiring different appropriate measures (Norris and Ortega, 2009; Lu, 2011; Bulté and Housen, 2014). Norris and Ortega (2009) proposed that to assess the syntactic complexity of L2 production systematically, researchers should incorporate measures for global complexity, complexity by subordination, complexity *via* sub-clausal or phrasal elaboration, and possibly complexity by coordination. Bulté and Housen (2014) examined the factors of time and genre together with the relationship to writing quality for a time span of one semester based on corpus essays, suggesting that the effects of time by using measures that detected change or development did not necessarily capture a higher level of language proficiency or better writing quality. Jiang et al. (2019) examined the syntactic complexity of 410 narrative compositions across four writing proficiency levels with both large-grained and fine-grained measures, aiming to arrive at certain indicators that could best discriminate and predict writing proficiency; they concluded that the mean length of production unit and the number of dependent clauses per clause could better predict writing proficiency than other traditional large-grained measures.

Since most of the previous studies on syntactic complexity were directed with limited measuring indices (Biber et al., 2011; Bulté and Housen, 2012), such as mean words per T-unit (W/T), words per clause (W/C), or dependent clause ratio (DC/C), it is necessary to measure syntactic complexity with a relatively comprehensive range of indices. Therefore, this study has adopted for analysis the 14 measures Lu (2011) proposed in the Syntactic Complexity Analyzer (SCA), including the six measures Ortega (2003) examined, a further five reviewed in Wolfe-Quintero et al. (1998), and another three recommended in Wolfe-Quintero et al. (1998) for further research. With the 14 measures examined on different aspects over one academic year, this study will be both insightful and informative as regards the developmental trajectories of syntactic complexity in argumentative and expository writing and also the effects exerted by the two genres.

### Previous studies on syntactic complexity in relation to genre differentiation

Measures of syntactic complexity have been proven to be sensitive to the genres of narrative and expository writing (Scott and Windsor, 2000; Beers and Nagy, 2009; Jeong, 2017) and those of narrative and argumentative writing (Crowhurst and Piche, 1979; Beers and Nagy, 2009; Lu, 2011; Qin and Uccelli, 2016; Yoon and Polio, 2016, 2017; Abdel-Malek, 2019; Jagaiah et al., 2020; Xu, 2020; Casal et al., 2021; Lu et al., 2021; Yu, 2021). Scott and Windsor (2000) found that students’ (9 and 11 years old) narratives had more clauses per T-unit than the expository summaries, while Crowhurst and Piche (1979) found that student (12 years old) narratives had fewer clauses per T-unit than persuasive essays (argumentative) and were not significantly different from the descriptive texts. Similarly, Ravid (2005) investigated two genres of L1 writing (narrative and expository) by writers ranging from fourth grade to adulthood and argued that expository texts, in which writers focused on abstract concepts and world knowledge, were more challenging to construct than narratives. The results revealed that children were likely to develop more complex language in expository texts despite the greater challenge posed by expository texts compared to narratives, which appeared less cognitively demanding.

Yoon and Polio (2017) examined narrative and argumentative essays written by 37 English-as-a-second-language (ESL) students to analyze development over time and genre differences. The results indicated strong genre differences in the area of linguistic complexity, with the syntactic complexity of argumentative essays being distinctly higher than that of narrative ones. In another study, Polio and Yoon (2018) investigated two automated systems, namely the L2 SCA and Coh-Metrix, as a way to capture variation in syntactic complexity across two genres. The results suggested that the syntactic complexity in argumentation was of greater magnitude when compared with that in narratives. Lu (2011) researched a corpus of college second language learners’ writing *via* the automatic SCA through examining 14 objective syntactic complexity measures to quantify second language writing development and progression. The results showed that the conditions of institution, genre, and time imposed a significant impact on the relationship between syntactic complexity and language performance, claiming that the clause was a potentially more informative unit of analysis than the T-unit. In the following year, Park (2012) examined the same 14 syntactic complexity measures as indices of language development in writing by college-level learners of English in Korea, also *via* SCA, revealing that genre made a great difference in regard to syntactic complexity in L2 learners’ writing, further confirming what had been found in Lu (2011).

With both genre and students’ proficiency taken into account, Beers and Nagy (2009) examined how syntactic complexity measures correlated with overall writing quality in two genres, narratives and expository essays. Their findings suggested that a clausal subordination measure (clauses per T-unit, C/T) was positively correlated with the quality of narratives but negatively with expository essays; conversely, there was no significant effect concerning the length of clause (W/C) on narrative writing, but statistical significance was found on expository writing. In another study, Beers and Nagy (2011) investigated the linguistic development of narrative, descriptive, compare/contrast, and persuasive compositions in different grade levels, and their results supported Crowhurst and Piche (1979) conclusion that narration places the fewest demands and argumentation the greatest demands on writers to make use of their syntactic resources.

Similarly, Yan and Zou (2019) examined differences in syntactic complexity in English among writers at two different proficiency levels and explored the relationship between syntactic complexity and compositions with two different genres. Compositions were also evaluated by SCA, gauging syntactic complexity at the global, clausal, and phrasal levels. The results indicated that the difference between the two genres reached a significant level in terms of C/T and complex nominals per clause (CN/C), but there was no significant relationship between syntactic complexity and L2 proficiency levels and no significant interactive effect between the genre factor and proficiency factor. Bae and Min (2020) investigated how syntactic patterns were different among four different genres, namely narrative, comparison, cause-effect, and argumentative, and three English proficiency levels in Korean L2 college students’ writing, examining the same 14 syntactic complexity measures as in Lu (2011). They found that syntactic complexity showed significant genre differences though no significant group differences of syntactic complexity were found among the L2 proficiency levels.

The findings yielded in previous studies have proven, to various degrees, the effects of genre on syntactic complexity in English writing, pointing to the different types of syntactic structures expected to be mastered in writing in different genres. However, the findings were mostly derived through comparisons between narratives and non-narratives and mostly between the narrative and argumentative genres. To fill the gap, the present study compares argumentative and expository compositions written by Chinese EFL learners over one academic year in an attempt to understand how genres affect syntactic complexities in EFL learners’ English writing and whether the genre effects are retained over 1 year.

## Methodology

### Research questions

1. What are the developmental changes in syntactic complexity over one academic year in Chinese EFL learners’ argumentative and expository compositions, respectively?
2. What are the effects exerted by genre on syntactic complexity in Chinese EFL learners’ argumentative and expository compositions, and are the effects of genre retained over one academic year?

### Tool and measures

Lu (2010) designed the SCA, which can automatically analyze the complexity measures of L2 writing. The SCA can yield data on 14 measures concerning length of production unit, amount of subordination, amount of coordination, and degree of phrasal sophistication (see Table 1). These 14 measures were previously employed or recommended in the two large-scale research syntheses by Wolfe-Quintero et al. (1998) and Ortega (2003). In 2011, Lu reported the results of his own study with SCA, with regard to syntactic complexity, as fair and independent measuring indices of university students’ writing based on a corpus of data. Park (2012) also evaluated the same 14 syntactic complexity measures, in a cross-sectional study, as valid indices of developmental patterns in writing by college learners of English in Korea. With a view to comparing the present study with Lu (2011) and Park (2012), we also adopted the same 14 syntactic complexity measures for analysis, which are further classified into five categories, to gauge the degree of syntactic complexity in Chinese students’ L2 argumentative and expository compositions.

**Table 1:** The five types of syntactic complexity measures examined in the present study.

Measure	Code	Definition
*Type 1: Length of production unit*		
Mean length of clause	MLC	Number of words divided by number of clauses
Mean length of sentence	MLS	Number of words divided by number of sentences
Mean length of T-unit	MLT	Number of words divided by number of T-units
*Type 2: Sentence complexity*		
Sentence complexity ratio	C/S	Number of clauses divided by number of sentences
*Type 3: Subordination*		
T-unit complexity ratio	C/T	Number of clauses divided by number of T-units
Complex T-unit ratio	CT/T	Number of complex T-units divided by number of T-units
Dependent clause ratio	DC/C	Number of dependent clauses divided by number of clauses
Dependent clauses per T-unit	DC/T	Number of dependent clauses divided by number of T-units
*Type 4: Coordination*		
Coordinate phrases per clause	CP/C	Number of coordinate phrases divided by number of clauses
Coordinate phrases per T-unit	CP/T	Number of coordinate phrases divided by number of T-units
Sentence coordination ratio	T/S	Number of T-units divided by number of sentences
*Type 5: Particular structures*		
Complex nominals per clause	CN/C	Number of complex nominals divided by number of clauses
Complex nominals per T-unit	CN/T	Number of complex nominals divided by number of T-units
Verb phrases per T-unit	VP/T	Number of verb phrases divided by number of T-units

### Participants

The current study was positioned in a 1-year-long *Comprehensive English* course, including *Comprehensive English III and Comprehensive English IV*, which was given to second-year English majors in a renowned university in Jiangsu province, China. The participants came from two parallel classes of the same grade, with 28 and 31 students, respectively. Of the 59 participants, 52 were girls and seven were boys, aged between 19 and 21, all with about 10 years of formal English learning experience. Moreover, all the courses, including both compulsory and optional ones, for the two classes were given by the same teachers. Because of the absence of a teacher working as an exchange scholar in the United States, the participants were put in the same class for the *Comprehensive English* course over the whole academic year, thus overcoming other potential intervening causes or variables. Finally, it should be pointed out that because some participants failed to complete and hand in all of the eight assigned compositions, only 52 (out of 59) participants’ compositions were valid for analysis in the present study.

### Data collection and analysis

Over the whole academic year, the participants were asked to write four argumentative and four expository compositions. The compositions were all finished in class, with a length of about 300 words and with no access to electronic devices. The writing topics were determined in relation to the contents of the textbook as well as the contemporary issues of the time. The data were collected at a regular time interval of 3–4 weeks, with the genres of argumentation and exposition alternated and the order of the topics counterbalanced to avoid a topic effect. To be specific, the topics for the first, third, fifth, and seventh compositions were argumentative, and those for the second, fourth, sixth, and eighth were expository (Table 2).

**Table 2:** The writing prompts for the argumentative and expository compositions.

Argumentative 1	Some people believe that the best way to learn about life is by listening to the advice of family and friends. Other people believe that the best way to learn about life is through personal experience. Which do you think is preferable? Use specific examples to support your preference
Expository 1	Many students have to live with roommates while going to school or university. What are some of the important qualities of a good roommate? Use specific reasons and examples to explain why these qualities are important
Argumentative 2	Some people learn best when a classroom lesson is presented in an entertaining, enjoyable manner. Other people learn best when a lesson is taught in a serious, formal way. Which of these two ways of learning do you prefer? Use specific reasons and details to support your choice
Expository 2	Every generation of people is unique in important ways. How is your generation different from your parents’ generation? Use specific reasons and examples to explain your answer
Argumentative 3	A growing number of people, especially the young, like eating at stands or restaurants, while many others prefer to prepare and eat food at home rather than eat out. Discuss both views and give your preference. Use specific reasons and examples to support your answer.
Expository 3	Imagine that you are preparing for a trip. You plan to be away from your home for a year. You need to make some preparations for the trip. What will you take? Explain why this thing/these things is/are important. Use specific reasons and details to support your choice(s)
Argumentative 4	For many university students there are two alternatives: one way is to find a job, the other is to pursue further study. Both have advantages and disadvantages, and it is difficult to say which is better. Discuss both views and then give your own opinion. Use specific reasons and examples to support your answer
Expository 4	People attend college or university for many different reasons (for example, new experiences, career preparation, and increased knowledge). Why do you think people attend college or university? Give reasons for your answer(s) and include any relevant examples from your own experience or knowledge

For each writing task, the scoring of the compositions was done by two experienced raters in accordance with the assessment criteria proposed for Chinese “FLTRP Cup” English writing contest in relation to content, organization, and language (Tian, 2014). However, the feedback given to the participants was concerned only with lexical and grammatical errors and not related to the use of the language structures required by different genres.

In the process of data collection, we first coded each of the compositions by all the participants into specific syntactic complexity indices *via* the L2 SCA (as specified in Table 1). For research question 1, we conducted a one-way ANOVA with time as the within-subjects variable, aiming to examine the developmental changes in syntactic complexities in argumentative and expository compositions, respectively. For research question 2, we first conducted a paired-sample T-test with genre as the within-subjects variable to compare the syntactic complexities of the first argumentative compositions and the first expository ones by the 52 participants written at the beginning of the academic year, the purpose of which was to see whether differences existed between argumentative and expository compositions. After that, comparisons were done consecutively between the second argumentative and second expository, the third argumentative and third expository, and the fourth argumentative and fourth expository compositions to see if the effects of genre had been retained over one academic year. Moreover, we conducted a two-way repeated measures analysis of variance with both genre and time as independent variables and the 14 measuring indices of syntactic complexity as dependent variables, aiming to explore the interactional effects between time and genre on developmental changes in syntactic complexity in argumentative and expository compositions.

## Results and discussion

### Changes in syntactic complexity in argumentative and expository compositions by Chinese EFL learners over one academic year

To see if there were any changes in syntactic complexity in Chinese EFL learners’ argumentative and expository compositions, a one-way ANOVA was conducted with time as the within-subjects variable in the two genres, respectively, as can be seen in Tables 3, 4.

**Table 3:** The effect of time on the syntactic complexity of argumentative compositions.

		Argumentative 1		Argumentative 2		Argumentative 3		Argumentative 4		*F*(3,204)	p
	Measures	*M*	*SD*	*M*	*SD*	*M*	*SD*	*M*	*SD*		
Length of production unit	MLC	9.183	1.378	10.434	1.627	10.444	1.978	10.533	1.649	7.747	0.000*
	MLS	19.039	4.219	20.875	4.745	19.228	4.377	19.811	4.606	1.758	0.156
	MLT	16.67	3.223	19.09	4.498	18.09	4.087	17.98	4.037	3.214	0.024*
Sentence complexity	C/S	2.093	0.457	2.009	0.378	1.856	0.349	1.887	0.366	4.107	0.007*
Subordination	C/T	1.829	0.3307	1.834	0.349	1.747	0.324	1.707	0.293	1.935	0.125
	CT/T	0.531	0.149	0.534	0.143	0.516	0.156	0.488	0.132	1.113	0.345
	DC/C	0.428	0.075	0.419	0.090	0.402	0.093	0.393	0.084	1.791	0.150
	DC/T	0.802	0.282	0.796	0.309	0.729	0.305	0.692	0.269	1.732	0.162
Coordination	CP/C	0.244	0.116	0.353	0.163	0.364	0.185	0.248	0.110	10.160	0.000*
	CP/T	0.44	0.203	0.640	0.305	0.621	0.315	0.420	0.187	10.494	0.000*
	T/S	1.145	0.139	1.099	0.099	1.065	0.086	0.107	0.117	4.401	0.005*
Particular structures	CN/C	0.989	0.259	1.220	0.330	1.16	0.310	1.310	0.370	9.228	0.000*
	CN/T	1.797	0.524	2.242	0.736	2.01	0.585	2.246	0.779	5.436	0.001*
	VP/T	2.517	0.492	2.624	0.609	2.68	0.507	2.617	0.524	0.841	0.473

* *p* < 0.05.

Not significant with Bonferroni adjustment.

**Table 4:** The effect of time on the syntactic complexity of expository compositions.

		Expository 1		Expository 2		Expository 3		Expository 4		*F*(3,204)	*p*
	Measures	*M*	*SD*	*M*	*SD*	*M*	*SD*	*M*	*SD*		
Length of production unit	MLC	9.82	1.74	10.118	1.564	9.785	1.238	10.607	1.818	2.914	0.035*
	MLS	17.772	4.34	19.107	5.198	18.859	4.262	19.765	3.949	1.795	0.149
	MLT	16.25	3.744	17.15	3.954	17.65	3.738	17.85	3.723	1.846	0.140
Sentence complexity	C/S	1.81	0.299	1.895	0.451	1.928	0.348	1.889	0.369	0.957	0.414
Subordination	C/T	1.657	0.277	1.699	0.317	1.803	0.282	1.67	0.285	2.378	0.071*
	CT/T	0.472	0.131	0.470	0.138	0.551	0.145	0.483	0.142	4.007	0.008*
	DC/C	0.379	0.079	0.369	0.095	0.412	0.079	0.383	0.087	2.406	0.068
	DC/T	0.646	0.246	0.653	0.278	0.761	0.262	0.672	0.258	2.151	0.095
Coordination	CP/C	0.259	0.151	0.308	0.122	0.276	0.118	0.342	0.166	3.537	0.016*
	CP/T	0.426	0.244	0.527	0.263	0.490	0.206	0.567	0.253	3.176	0.025*
	T/S	1.095	0.088	1.112	0.112	0.070	0.089	0.114	0.127	1.919	0.128
Particular structures	CN/C	1.13	0.230	1.14	0.250	1.010	0.250	1.160	0.340	10.968	0.000*
	CN/T	1.895	0.558	1.953	0.571	1.846	0.621	2.208	0.625	3.833	0.011*
	VP/T	2.359	0.446	2.261	0.510	2.575	0.540	2.481	0.482	4.002	0.009*

* *p* < 0.05.

Not significant with Bonferroni adjustment.

Of the 14 measures examined, there was a linear growth in MLC in both argumentative and expository compositions over time, which concurs with the findings yielded in previous studies (Wolfe-Quintero et al., 1998; Xu et al., 2013). The significant and positive increase in MLC and MLT in argumentative compositions and MLC in expository compositions indicated students’ increased ability to produce longer expressions. Moreover, significant increase was also found in CP/C, CP/T, CN/C, and CN/T in both argumentative and expository compositions. Although the production of longer units does not necessarily equal complex and good language, it demonstrates the writer’s ability to form them in one sentence or clause by connecting different views and expressions, and the ability to master and use longer clauses and sentences does, to some extent, exhibit a higher level of language proficiency (Beers and Nagy, 2011). On one hand, we may attribute the longer mean length of production unit to the use of more clauses; on the other hand, the production of longer language units can be the result of phrasal elaboration. The increased values of CP/C, CP/T, CN/C, and CN/T in both argumentative and expository compositions suggest that complex phrases, such as attributive phrases, appositive phrases, and adjective phrase, as well as complex nominal expressions, can be used as a good way to increase the length of language production (Ansarifar et al., 2018).

It is interesting to see that the first argumentative and first expository compositions both had a relatively higher value of C/T, which means that more clauses were incorporated into the sentences in the students’ language production. However, the value of C/T experienced a drastic drop in both argumentative and expository writing over the course of the year, which concurs with Ortega (2015) view that as language learners advance to a higher level, they are more likely to produce language that is typically characterized by the use of phrasal expressions rather than progress at the clausal level. The development of coordination and particular structures in both argumentative and expository compositions, and meanwhile the decreased value of subordination in argumentative and minor increase in expository, indicated that the L2 writers moved toward the development of more phrasal components as compared to clausal components as they advanced in writing (Crossley and McNamara, 2014). This finding further corroborates Biber et al. (2011), who claimed that syntactic complexity could be better measured with the measuring indices of noun phrases rather than clauses embedded in sentences and that in writing academic essays, meaning was mostly expressed through the use of complex noun phrases rather than clause-level expressions. Moreover, the developmental trend concluded in the present study is in line with the developmental sequence proposed by Norris and Ortega (2009), where more advanced stages of development were defined by the extended use of nominalization. Likewise, Biber et al. (2013) proposed that the developmental stage of L2 learners is similar to the sequence theory and that there are specific developmental progressions in which grammatical form develops from finite dependent clauses to non-finite dependent clauses and to dependent phrases, and on the other hand, learners’ syntactic function progresses from the use of clausal constituents (e.g., direct object or adverbial) to the use of noun phrase modifiers (Biber and Conrad, 2009; Biber and Gray, 2010; Biber et al., 2013).

### Effects of genre on syntactic complexity in Chinese EFL learners’ writing

To answer the second research question, the paired-sample *t*-test was first conducted with genre as the within-subjects variable between the first argumentative and the first expository compositions, and then comparisons were made, consecutively, between the second argumentative and expository compositions, the third argumentative and expository compositions, and the fourth argumentative and expository compositions on all of the 14 measures. Table 5 presents the statistically significant differences between syntactic complexities in argumentative and expository compositions at four time points over the year.

**Table 5:** Mean differences (MD) in syntactic complexities and standard deviations by time point and genre.

		Time 1 (Arg.1 and Exp.1)			Time 2 (Arg.2 and Exp.2)			Time 3 (Arg.3 and Exp.3)			Time 4 (Arg.4 and Exp.4)		
	Measure	MD	*t*	*p*	MD	*t*	*p*	MD	*t*	*p*	MD	*t*	*p*
Length of production unit	MLC	−0.637	−2.585	0.013*	0.316	1.099	0.277	0.659	2.649	0.001*	−0.074	−0.348	0.729
	MLS	1.266	3.153	0.003*	1.767	2.979	0.004*	0.369	0.682	0.499	0.047	0.082	0.935
	MLT	0.429	1.115	0.270	1.936	3.850	0.000*	0.445	0.835	0.407	0.130	0.236	0.814
Sentence complexity	C/S	0.283	4.466	0.000*	0.114	1.802	0.078	−0.072	−1.422	0.161	−0.002	−0.031	0.975
Subordination	C/T	0.171	3.248	0.002*	0.134	2.694	0.010*	−0.055	−1.144	0.258	0.010	0.189	0.851
	CT/T	0.059	2.643	0.011	0.064	2.916	0.005*	−0.036	−1.410	0.165	0.005	0.228	0.820
	DC/C	0.048	3.641	0.001*	0.050	3.302	0.002*	−0.010	−0.662	0.511	0.009	0.553	0.583
	DC/T	0.155	3.232	0.002*	0.143	3.229	0.002*	−0.032	−0.679	0.500	0.019	0.389	0.699
Coordination	CP/C	−0.015	−0.868	0.389	0.044	1.900	0.063	0.088	3.061	0.003*	−0.094	−4.502	0.000*
	CP/T	0.014	0.455	0.651	0.113	2.888	0.006*	0.132	2.747	0.008*	−0.147	−4.249	0.000*
	T/S	0.050	2.558	0.014*	−0.012	−0.628	0.533	−0.005	−0.370	0.713	−0.007	−0.363	0.718
Particular structures	CN/C	−0.146	−3.906	0.000*	0.079	1.558	0.126	0.149	3.598	0.001*	0.162	3.916	0.001*
	CN/T	−0.097	−1.253	0.216	0.289	2.950	0.004*	0.164	2.040	0.004*	0.038	0.378	0.706
	VP/T	0.158	2.191	0.033*	0.363	4.684	0.000*	0.105	1.471	0.147	0.136	1.542	0.002*

* *p <* 0.05.

Not significant with Bonferroni adjustment.

Table 5 shows that the values of the argumentative compositions were significantly different from those of the expository ones written by the participants at Time 1. To be precise, on the length of the production units, MLC [*t*(−2.585), *p* = 0.013] and MLS [*t*(3.153), *p* < 0.05] were found to be of statistical significance; C/S [*t*(4.466), *p* = 0.000] was also of statistical significance concerning the effect of genre; on the level of subordination, C/T [*t*(3.248), *p* = 0.002], DC/C [*t*(3.641), *p* = 0.001], and DC/T [*t*(3.232), *p* = 0.002] were significantly and positively related to genre; on sentence coordination, T/S [*t*(2.558), *p* = 0.014] was the only measure that was of significance on the level of coordination; for the measuring indices in particular structures, a positive statistical significance was detected on VP/T [*t*(2.191), *p* = 0.033]. Inspections of the two group means indicate that learners produced overall more complex language in argumentative compositions than expository ones at Time 1 except for the negative relation on the measures of MLC [*t*(−2.585), *p* = 0.013] and CN/C [*t*(−3.906), *p* = 0.000]. Similar to the results yielded between the first argumentative and first expository compositions, at Time 2, significant effects were found on the length of the production units MLS [*t*(2.979), *p* = 0.004] and MLT [*t*(3.850), *p* = 0.000]; all the measures of subordination, namely C/T [*t*(2.6945), *p* = 0.010], CT/T [*t*(2.916), *p* = 0.005], DC/C [*t*(3.302), *p* = 0.002], and DC/T [*t*(3.009), *p =* 0.002], were found to be positively and significantly related to genre; CP/T [*t*(2.888), *p* = 0.006] was the only measure that was detected to be of significance on coordination measures; for particular structures measures, the differences concerning CN/T [*t*(2.950), *p* = 0.004] and VP/T [*t*(4.684), *p* = 0.000] were of statistical significance. Simply put, at Time 1 and Time 2, the genre effects and statistical significance were found mostly on the length of production unit, sentence complexity, and subordination.

However, at Time 3, the statistically significant differences were found more on coordination and particular structures. The results obtained at Time 4 were similar to those at Time 3. Put another way, for Time 3 and Time 4, the measures of coordination and particular structures were found to be significantly and positively related to genre. To be precise, at Time 3, there were significant genre effects on the measures of CP/C [*t*(3.061), *p* = 0.003], CP/T [*t*(2.747), *p* = 0.008], CN/T [*t*(2.040), *p* = 0.046] and CN/C [*t*(3.598), *p* = 0.001]. At Time 4, the genre effects were found on the measures of CP/C [*t*(−4.502), *p* = 0.000], CP/T [*t*(−4.249), *p* = 0.000], CN/C [*t*(3.916), *p* = 0.001], and VP/T [*t*(1.542), *p* = 0.002].

Through comparing the syntactic complexities in argumentative and expository compositions at four time points, we can conclude that the syntactic complexities of both argumentative and expository compositions in the earlier stage (the first semester in our research) were exhibited more on the measures of subordination and length of production unit, which indicated clausal progression, whereas the later period (the second semester in our research) saw a development of syntactic complexity on the measures of coordination and particular structures, both of which showcased the development of phrase-level complexity.

Moreover, the two-way repeated measures ANOVA was also conducted with genre and time as independent variables and the 14 measures of syntactic complexity as dependent variables (shown in Table 6). Along with the exact p-values, this study also reported the effect sizes and partial eta squared (ηp^2^), an appropriate measure for research designs involving within-subjects factors. Regarding the role that time played on the developmental pattern of syntactic complexity, the findings reveal that there was a significant main effect for time mainly on the measures of the length of production unit, namely MLC (*p* = 0.000, ηp^2^ = 0.055), MLT (p = 0.008, ηp^2^ = 0.029), and MLS (*p* = 0.043, ηp^2^ = 0.020), indicating that L2 writers produced longer expression in argumentative compositions than in expository compositions over the course of the year. On the coordination level, the significance was on all the three measures, namely CP/C (*p* = 0.000, ηp^2^ = 0.042), CP/T (*p* = 0.000, ηp^2^ = 0.048), and TS (*p*=0,003, ηp^2^ = 0.031). And similarly, all the three measures on the particular structures were found to be significantly related to time: CN/C (*p* = 0.000, ηp^2^ = 0.100), CN/T (*p* = 0.000, ηp^2^ = 0.053), and VP/T (*p* = 0.021, ηp^2^ = 0.023), showing that L2 learners tended to use more phrases in their compositions to express meaning and connect ideas. Different from the role that time played on syntactic complexity, the effect of genre was mainly found to be on the measures of the length of production unit (MLS: *p* = 0.050, ηp^2^ = 0.009; MLT: *p* = 0.055, ηp^2^ = 0.009), sentence complexity (C/S: *p* = 0.031, ηp^2^ = 0.011) and subordination (C/T: *p* = 0.032, ηp^2^ = 0.011, DC/C: *p* = 0.004, ηp^2^ = 0.020, and DC/T: *p* = 0.009, ηp^2^ = 0.017), and VP/T (*p* = 0.000, ηp^2^ = 0.034) of particular structures. There were interactive effects of time and genre, and statistically significant difference was found on MLC (*p* = 0.031, ηp^2^ = 0.022), C/S (*p* = 0.005, ηp^2^ = 0.031); all the measures of subordination, namely C/T (*p* = 0.027, ηp^2^ = 0.022), CT/T (*p* = 0.034, ηp^2^ = 0.021), DC/C (*p* = 0.026, ηp^2^ = 0.022), and DC/T (*p* = 0.035, ηp^2^ = 0.021)); CP/C (*p* = 0.000, ηp^2^ = 0.054) and CP/T (*p* = 0.000, ηp^2^ = 0.047) in coordination; and CN/C (*p* = 0.003, ηp^2^ = 0.034) of particular structures.

**Table 6:** Genre effects on syntactic complexity measures (two-way repeated measures ANOVA).

		Genre		Time		Genre*Time	
	Measures	*p*	ηp^2^	*p*	ηp^2^	*p*	ηp^2^
Length of production unit	MLC	0.682	0.000	0.000*	0.055	0.031*	0.022
	MLS	0.050*	0.009	0.043*	0.020	0.485	0.006
	MLT	0.055	0.009	0.008*	0.029	0.334	0.008
Sentence complexity	C/S	0.031*	0.011	0.438	0.007	0.005*	0.031
Subordination	C/T	0.032*	0.011	0.315	0.009	0.027*	0.022
	CT/T	0.098	0.007	0.102	0.015	0.034*	0.021
	DC/C	0.004*	0.020	0.362	0.008	0.026*	0.022
	DC/T	0.009*	0.017	0.416	0.007	0.035*	0.021
Coordination	CP/C	0.682	0.000	0.000*	0.044	0.000*	0.054
	CP/T	0.259	0.003	0.000*	0.052	0.000*	0.047
	T/S	0.547	0.001	0.003*	0.033	0.134	0.014
Particular structures	CN/C	0.529	0.001	0.000*	0.100	0.003*	0.034
	CN/T	0.112	0.006	0.000*	0.053	0.147	0.013
	VP/T	0.000*	0.034	0.021*	0.023	0.262	0.010

* *p* < 0.05.

Not significant with Bonferroni adjustment.

While comparing the findings from the present study with those obtained in previous research, we found that the adoption of different measuring indices may lead to different results in different types of genres. Nonetheless, most previous studies focused only on a few measures in contrasting narrative essays and non-narrative ones, and therefore it is difficult and unpersuasive in regard to determining if the indices taken are representative enough to capture the differences or whether there are other measures that are equally persuasive in demonstrating syntactic complexity.

The findings yielded in the present study are consistent with those obtained in Beers and Nagy (2009), who examined three measures, namely C/T, W/C, and W/T, and ascertained genre differences when using W/T and C/T as measures of syntactic complexity. Moreover, our results were comparable to those of Lu (2011), who found higher complexity measures in argumentative essays than in narratives under the condition of controlling for both time and institution on the measure of length of production unit (MLS).

On the whole, the majority of measures of statistically significant difference concerning genre effects were detected on the clausal level though the effects of time were exhibited more on the length of production unit, on coordination, and on particular structures, the last two of which being measures featuring the progression of phrase-level expressions. We may safely conclude that participants’ language production was affected by the type of writing tasks assigned to them and that between the two non-narrative types of writing, argumentative compositions exhibited higher syntactic complexity than expository compositions, which is in line with previous findings (Bulté and Housen, 2014; Kyle and Crossley, 2018). Put another way, argumentative compositions placed more reasoning demands on participants than expository ones, thus resulting in higher syntactic complexity.

Looking at the data in detail, we found that in the first semester (Time 1 and Time 2), the measuring indices were shown to discriminate more on subordination and the length of production unit, both of which were on the clausal level, despite the few measures found to be significantly related in coordination and particular structures. At Time 3 and Time 4, the effects of genre were found to be more on coordination and particular structures. This finding was consistent with the results reported in Lu (2011) and Xu et al. (2013) but partly inconsistent with the results that Ortega (2003) drew. Ortega ascribed the relatively small magnitude of C/T to advanced learners being more likely to simplify the clauses to shorter phrases and supported the claim that C/T may be lower at advanced levels as a result of reduction from clauses to phrases (2003). Lu (2011) found that for clauses per T-unit, significant differences were detected within persuasive compositions, in which there were more clausal-level expressions concerning subordination; whereas in our study, the significance was found in expository compositions rather than in argumentative ones. And for W/C (the corresponding index in our study is MLC), there were more W/C in descriptive compositions than in persuasive ones, but a statistically significant difference was not detected in the other two genres of writing. This was partly inconsistent with our study, in which the argumentative essays demonstrated longer lengths of clauses, with participants producing longer clauses in argumentative than in expository compositions.

To probe deeper into the causes underlying the difference, we excerpted some of the argumentative and expository compositions in the following:

Argumentative writing:

Student A: The booming catering industry has been capturing the hearts of more and more people especially the young, who like eating at stands or restaurants. Anyway, many others make a point to prepare and eat at home. Those who have good reasons to eat out argue that cooking at home wastes time as well as energy. With exhausting or maybe dull routine work, they do not bother to cook anymore. In addition, it seems that cuisine is more palatable and multiple at diners or restaurants, where they can just wait and soon enjoy the meals.

Student B: However, it’s not the only access to earn more and get promoted. To new graduates, finding a job earlier means more working experience and larger circle of professional friends, from whom they could seek supports when tackling challenges. They also have an edge over master degree (or up) holders in good energy. People in their mid-twenties would be more concentrated in working for they have little affairs to cope with in terms of family and seldom do they have to take a sick leave, compared with some middle-aged people.

Expository writing:

Student A: I’m preparing for long relaxing, a solo travel which will last for one year. I want to feel the romance of European towns, the spectacle of African savannah and the vast of the ocean. However, the luggage is an important matter which will affect my aesthetic experience. It cannot be too much or lacking. Therefore, besides all kinds of clothes and the cellphone used to pay, contact family and friends and take photos, I am to pick out following necessary articles.

Student B: Now as a college student, what I have learned from little experience in school is that the life here prepares for the life later. I entered XXX university last year and tried all kinds of activities I had never tried before, such as working as a volunteer in various corners of the city, participating in school organizations and staying up all night in the study room. I learned how to utilize my kindness to benefit people in need and effectively cooperate with partners. I realized that nobody can succeed with no venture. Thus I would push myself harder.

In analyzing the excerpts of participants’ compositions, we can see that, compared with expository compositions, there are more clauses in participants’ argumentative compositions, such as attributive clauses (…*the young*, *who like eating at stands…*), adverbial clauses (…*they could seek supports when tackling challenges*), and predicative clauses (*it seems that cuisine is*..). The use of more clauses in argumentative compositions, for one thing, discriminates the value of MLC with that of expository ones, and for another, it partly explains the high magnitude of C/T observed in our study. As with more clauses incorporated into a sentence, it is not surprising that the value of C/T gets higher accordingly. As opposed to more clauses found in argumentative compositions, in expository writing, there were more phrasal-level expressions, such as *the romance of European towns*, *the spectacle of African savannah*, *the vast of the ocean*, *working as a volunteer in various corners of the city*, *participating in school organizations*, and *staying up all night in the study room*.

In previous studies, researchers found that expository texts composed by student writers have more complex noun phrases (Ravid and Berman, 2010), more nominalized forms (Schleppegrell, 2001), and more relative and adverbial clauses (Scott and Windsor, 2000) as opposed to narratives, which suggests that the narrative and expository genres require different cognitive demands. The inherently different nature of narrative and non-narrative compositions, and even different types of writing within non-narrative ones, indicates that certain types of texts are believed to be linguistically and cognitively more demanding than others (Schleppegrell, 2001), and the frequency of exposure to different communicative contexts differs remarkably across individuals, leading to overall different structures and different levels of syntactic complexity. In the same vein, learners’ language production of argumentative and expository writing in syntax is characterized by distinctive forms and expressions. As Beers and Nagy (2009) stated, “writers need to be aware of stylistic options that will produce the most desirable impression on their readers most. Among these stylistic options are a variety of sentence-level syntactic structures through which meaning can be conveyed” (p. 196).

Moreover, in comparing the changes in syntactic complexity of both argumentative and expository writing, we found a positive progression in both genres of writing over the course of the year in most of the measures. But the developmental pattern and mean value of the syntactic complexity measures of argumentative and expository compositions are different. As for the mean length of production unit, all the three measures showed a generally upward trend with minor fluctuation. And the mean value of the MLC of argumentative compositions was consistently higher than that of expository ones despite the progression of both genres, and so was the syntactic complexity of MLS and MLT. As for other clause-level measures, like subordination, we see a generally decreased value of syntactic complexity in both genres of writing (although no significance was detected), which confirms previous findings that as learners advance to higher levels, they learn to use complex phrases rather than progress in clausal expressions (Ortega, 2003). Unlike the previous measuring indices, the four measuring indices on coordination behave differently in that students achieved more notable progress on CP/T and CP/C in expository writing than in argumentative writing and that the magnitude of C/T experienced a drastic decrease over the academic year. The relatively higher magnitude of C/T at the beginning was probably because when the students were trying to express the relationship among ideas, they were more likely to use more clauses to make their expressions smooth and coherent, such that more clauses were contained in each T-unit (Ortega, 2003). The progression pattern corroborated with other measures on the last set of indices, the particular structures. We found that all the three measures’ value increased value and that two of the measures (CN/C and VP/T) distinguished argumentative writing from expository writing positively and significantly.

To conclude, of the 14 measures we used to quantify syntactic complexity, it is clear that argumentative compositions and expository compositions are distinctively and significantly different from each other and that learners generally achieved more complex language production in both genres of writing over one academic year. Looking at the interactive effects of both time and genre, in spite of the progression of students’ writing in both genres, we found that the syntactic complexity of argumentative compositions was still higher than that of expository ones for the majority of measures and for most of the time points. In Biber and Conrad (2009) explanation, this was due to the fact that different types of writing entail distinct functional requirements, which could result in learners’ different language production. Moreover, argumentative writing involves more complex functional demand, and therefore the resources needed for completing it can be more than those required for expository writing. By looking at the developmental trend of the 14 measures over 1 year, we found that the linguistic progression or development proceeded in a similar, if not the same, way across the two genres, which is in line with our hypothesis concerning the syntactic complexity difference. In light of Robinson (2007) framework, we can attribute the difference between the two types of writing to the cognitive demand, which states that increased cognitive complexity (in our study, argumentative writing) imposes great effects on language production. While performing argumentative writing, which is a more complex task with more demands of logical and causal reasoning, participants tended to draw on their attention and memory resources to meet the requirements of more demanding cognitive resources and therefore produced language of relatively higher complexity. More importantly, time and instruction imposed essentially little influence on the linguistic progression between argumentative and expository writing. Therefore, we may safely attribute the different levels and progressions of syntactic complexity in argumentative and expository compositions to the inherently different natures and demands of the two genres of writing, which verifies our hypothesis that the distinctive cognitive resources required by the two types of writing genres play a crucial role in students’ writing syntactic complexity.

There is one more point worthy of special note in our study. In reading through all the argumentative and expository compositions, we found that when participants were required to produce expository compositions, some of them wrote by explaining their reasons and relating those reasons to instances for discussion. And they imposed a logical structure to interrelate ideas in a coherent manner and to organize claims and arguments in a stepwise hierarchical format, which is a typical way of organizing argumentative writing. We are not sure whether it was the organization of their expository compositions in an argumentative way that led to the relatively high value of C/T in the expository compositions, but we do know that some students were not quite aware of the requirements of different structures pertinent to different genres. Therefore, teaching EFL should not be understood as promoting a global English proficiency expected to be equally functional across different genres, but rather specific and genre-targeted instruction should be taken into consideration in the actual learning and teaching.

## Conclusion

The findings of the present study are twofold. On one hand, we found that there was a statistically significant development of syntactic complexity in both argumentative and expository compositions and that the development was found more in phrasal-level measures (coordination and particular structures) than in clausal ones. This finding concurs with Biber and Gray (2010) that complex noun phrases can be much more appropriate measures of syntactic complexity compared to embedded clauses. It also echoes Biber et al. (2013), who highlighted the criticality of considering the distinctive grammatical features of academic register, i.e., phrasal modifiers when conducting syntactic complexity analysis in L2 writing research. On the other hand, genre was found to have affected the development of syntactic complexity in argumentative and expository compositions. To be precise, the syntactic complexity of argumentative essays was significantly higher than that of expository essays in some of the measuring indices, and this finding corroborates the findings of previous studies conducted between narrative and non-narrative genres (Beers and Nagy, 2011; Yoon and Polio, 2017), indicating that even within non-narrative types of writing, significant differences still exist. Moreover, the effects of genre were retained over one academic year, thus confirming the importance of controlling for the effects of relevant learner-, task-, and context-related factors in interpreting between-proficiency differences in syntactic complexity (Lu, 2011), and therefore conflating non-narratives (argumentative and expository in the present study) may overlook these differences.

The implications of the study are also twofold. First, the present study may help EFL learners and writing instructors gain a more in-depth understanding of how and in what forms the features of syntactic complexity are demonstrated. The lack of clause-level statistically significant development in both argumentative and expository essays may indicate that academic writing is characteristically dense with non-clausal phrases and complex noun phrases and that therefore phrases may be taken as more appropriate measures of syntactic complexity as opposed to embedded clauses. In academic prose, meaning is, for the most part, condensed into complex noun phrases rather than being expressed in clause forms, and therefore noun phrase modification, such as attributive adjectives and post-noun-modifying prepositional phrases, has a tendency to contribute to essay quality, which requires researchers and teachers to re-examine the traditional focus on clausal embedding and subordination measures based on the assumption that academic writing derives its complexity from the elaborate use of clausal construction and, for another, provides possible ways for them to place emphasis on the learning and application of learners’ phrasal-level knowledge. Second, learning a language can be taken as learning a range of distinct types of discourses, which are context-specific (Qin and Uccelli, 2016), and the knowledge and ability acquired in one particular genre practice does not necessarily mean that students can transfer their successful performance from one genre to another. Therefore, English teaching cannot be taken just as a way to promote students’ overall language level and performance, which is generally believed to serve the same function in different contexts, and students should also be able to draw inferences from one another. Both teachers and researchers should reflect on the different needs students will encounter in authentic communicative contexts in order to make informed decisions concerning which discourse practice to select for instruction. In the meantime, when assessing students’ language performance, especially in different genres of writing tasks, the effects of genre on students’ language production should also be taken into consideration since different genres of writing may entail specific syntactic expressions, and the progression of each genre can be distinct.

As with most studies, ours was not without its limitations. In the first place, the participants in this study were all from the same class and the same grade, leading to relatively high homogeneity, and so whether the findings of this research are generalizable needs to be further verified. Future research could involve subjects from different language proficiency levels, such as participants from different grades, institutions, and even different L1 backgrounds to investigate the syntactic development in students’ writing with language level and genre as interactive factors. Also, comparing results from EFL learners with those of native speakers may generate more robust findings concerning the effects of genre on syntactic complexity. Moreover, although one academic year is not considered short in regard to collecting data, it is more desirable to conduct research over a longer time span. Additionally, we adopted the 14 measures that Lu (2011) used for SCA, and it is undeniable that syntactic complexity includes various sorts of linguistic features, such as discourse features, passive sentences, and other linguistic resources used to distinguish one’s own position from that of others. Therefore, it would also be useful to employ more comprehensive and fine-grained measures for comparison. However, it is worth noting that in quantifying syntactic complexity, it is not the case that the more, the better, and therefore choosing indicators that represent a genuine and comprehensive measure of syntactic complexity matters most.

## Data availability statement

The original contributions presented in the study are included in the article/supplementary material, further inquiries can be directed to the corresponding author.

## Ethics statement

The studies involving human participants were reviewed and approved by School of Foreign Languages, Soochow University. The patients/participants provided their written informed consent to participate in this study.

## Author contributions

LP: conceptualization, research design, data analysis, and manuscript revision. RH: research design, data analysis, drafting, and manuscript revision. CC: data collection and data analysis. All authors contributed to the article and approved the submitted version.

## Conflict of interest

The authors declare that the research was conducted in the absence of any commercial or financial relationships that could be construed as a potential conflict of interest.

## Publisher’s note

All claims expressed in this article are solely those of the authors and do not necessarily represent those of their affiliated organizations, or those of the publisher, the editors and the reviewers. Any product that may be evaluated in this article, or claim that may be made by its manufacturer, is not guaranteed or endorsed by the publisher.
